# Motoneuron firing in amyotrophic lateral sclerosis (ALS)

**DOI:** 10.3389/fnhum.2014.00719

**Published:** 2014-09-22

**Authors:** Mamede de Carvalho, Andrew Eisen, Charles Krieger, Michael Swash

**Affiliations:** ^1^Institute of Physiology and Institute of Molecular Medicine, Faculty of Medicine, University of LisbonLisbon, Portugal; ^2^Department of Neurosciences, Hospital Santa Maria, Faculty of Medicine, University of LisbonLisbon, Portugal; ^3^Emeritus Professor of Neurology, University of British ColumbiaVancouver, BC, Canada; ^4^Department of Biomedical Physiology and Kinesiology, Simon Fraser University, BurnabyBC, Canada; ^5^Department of Medicine (Neurology), University of British Columbia, VancouverBC, Canada; ^6^Institute of Neuroscience, Barts and The London School of Medicine, Queen Mary University of LondonLondon, UK

**Keywords:** amyotrophic lateral sclerosis, lower motor neuron, motor cortex, motor units firing, upper motor neuron

## Abstract

Amyotrophic lateral sclerosis is an inexorably progressive neurodegenerative disorder involving the classical motor system and the frontal effector brain, causing muscular weakness and atrophy, with variable upper motor neuron signs and often an associated fronto-temporal dementia. The physiological disturbance consequent on the motor system degeneration is beginning to be well understood. In this review we describe aspects of the motor cortical, neuronal, and lower motor neuron dysfunction. We show how studies of the changes in the pattern of motor unit firing help delineate the underlying pathophysiological disturbance as the disease progresses. Such studies are beginning to illuminate the underlying disordered pathophysiological processes in the disease, and are important in designing new approaches to therapy and especially for clinical trials.

## INTRODUCTION

Motor unit recruitment and firing is fundamental to voluntary movement and motor control. Adrian and Bronk (1929) studied motor unit recruitment and firing rates in normal human muscle, using their newly fabricated concentric needle electrode. They noted that “force exerted by a muscle during a voluntary contraction was the result of the concurrent recruitment of motor units and modulation of the rate at which they discharged action potentials.” Denny-Brown and Pennybacker (1938), observed that in the process of recruitment of motor units in electromyographic (EMG) recordings the first recruited units were always small units, successively larger units being recruited as force increased. Henneman et al. (1965), confirmed a pattern of orderly recruitment of successively larger motor units, as deduced by studies of action potentials recorded from motor nerves, not muscle, and introduced the concept of the “size principle” (Grimby and Hannerz, 1968, 1981; Duchateau and Enoka, 2011), showing that at lower levels of activation only low-threshold slow-fatiguing motor units are recruited. At higher force requirements motor units fire more rapidly (Denny-Brown and Pennybacker, 1938). The technological limitations imposed by electrode recording characteristics in human EMG recordings during recruitment imply that low threshold units are sampled almost exclusively in EMG recordings (Grimby and Hannerz, 1981). Grimby and Hannerz (1981), also suggested that, during phasic activity, motor units are recruited in a different order than in tonic activity. More recently, Stein et al. (1972), and Milner-Brown et al. (1973), confirmed that motor units were recruited in relation to size.

Studies of motor unit firing have been neglected in categorizing lower motor neuron (LMN) and upper motor neuron (UMN) disorders, and in mixed UMN and LMN disorders, for example amyotrophic lateral sclerosis (ALS; de Carvalho et al., 2012).

## UPPER MOTOR NEURON IN AMYOTROPHIC LATERAL SCLEROSIS

The modulation of spinal motor neuron firing by the brain and spinal pathways via UMNs, especially by the motor cortex, poses a particular problem in motor physiology, and in understanding the clinical effects of lesions in the motor system (Denny-Brown, 1966). ALS is a motor system disease, although extra-motor areas are also involved (van der Graaff et al., 2009). Magnetic resonance imaging (MRI) analysis of regional volumetric changes in ALS patients, particularly voxel-based morphometry (VBM), has indicated that brain atrophy occurs not only in motor areas, but also in non-motor areas, including frontal, temporal, and parietal lobes of both hemispheres (Agosta et al., 2010; Turner and Modo, 2010; Mezzapesa et al., 2013). Thus ALS is a degenerative brain disease, which is not confined to the motor system. Frontotemporal Dementia (FTD), is a well established, disorder having some features similar to ALS (Lillo and Hodges, 2009; Neary and Snowden, 2013), an observation underscored by the discovery of a hexanucleotide repeat expansion in the first intron of the C9ORF72 gene on chromosome 9p21 associated with both ALS and FTD (DeJesus-Hernandez et al., 2011; Renton et al., 2011). In addition, evolutionary and early life developmental concepts as they relate to the clinical deficits in ALS, also support early cortical involvement in ALS (Eisen et al., 2013).

Classic Charcot-type ALS is characterized by a variable combination of upper and LMN deficits. In about 1% of ALS patients, the UMN component appears in isolation for many years (by definition for more than 4 years); this is referred to as primary lateral sclerosis (PLS). In about 10% of patients there are isolated LMN features, termed progressive muscular atrophy (PMA). Pathological studies reveal that there is corticospinal (CST) tract degeneration in both PLS and PMA (Ince et al., 2003), an observation that led Gowers (1893) to classify PLS, PMA, and ALS as variants of a single clinical entity, later classified by Brain (1962) as Motor Neuron Disease. PLS usually presents insidiously in the sixth decade of life, with a symmetric, slowly progressive spastic paresis, beginning in the lower extremities, that evolves into a tetrapyramidal syndrome with marked pseudobulbar features (Pringle et al., 1992). Most patients with PLS later develop LMN features. Resting state functional MRI changes in patients with PLS resemble those of ALS (Agosta et al., 2014).

### SITE OF ONSET OF ALS

When Charcot (1874) coined the term ALS, he recognized the importance of degeneration of the lateral columns and of cells in the ventral horns of the spinal cord, either occurring together, which he termed deuteropathic change or only involving only the ventral horns, which he termed protopathic. The notion of upper or LMN syndromes was then unknown, although Lockhart Clarke, in London (Turner et al., 2010), had shown that loss of cells in the ventral horns was accompanied by muscular atrophy. No observations were made as to the possible relevance of these observations to causation of ALS. In his Tuesday lectures, Charcot laid stress on “fibrillary twitches” of muscles in ALS as a particular feature of the disease. Gowers (1893), pointed out that ALS seemed to begin focally and then to spread through the motor system. In contemporary studies, the earliest changes in ALS have been studied using transcranial magnetic stimulation (TMS; Vucic et al., 2013; Vucic and Kiernan, 2013), and functional MRI (Foerster et al., 2013; Zhang et al., 2014). Although there remains uncertainty as to whether “sick” corticomotor neurons can induce anterograde death of spinal motoneurons in ALS, or if corticomotoneurons and anterior horn cells degenerate independently, as suggested in PLS or PMA (Eisen and Weber, 2001), the current evidence supports early cortical motor hyperexcitability in ALS. The importance of the degeneration of cortico-motoneuronal cells in the early clinical findings of loss of dexterity in ALS is well recognized. Indeed, a relatively small reduction of these motoneurons will have greater clinical impact than loss of cortical neurons involved in simpler motor functions (Eisen and Weber, 2001).

### MOTOR CORTEX (M1) NEURONS IN ALS

Precise control of fine finger movement is a characteristic of human motor behavior. The motor neuron circuitry, which includes neurons and cells located both in the cerebral cortex and the spinal cord, is controlled by a complex neural network (Jara et al., 2014). This initiates precise movement of the legs, arms, and hands, breathing, and vocalization, all of which become severely compromised in ALS. The cortical motoneurons known as Betz cells in humans are located in layer V of the motor cortex. They are also referred to as, CST neurons, or corticomotoneurons. These neurons are characterized by: (1) a large pyramidal cell body, (2) a single apical dendrite that extends toward layer I displaying major branching and arborization, especially within layer II/III, (3) numerous basal dendrites arising from the basolateral surface, and (4) a very long axon that projects toward spinal cord targets (Molnar and Cheung, 2006; Ozdinler and Macklis, 2006; Molyneaux et al., 2007).

Corticospinal neurons are distributed over broad regions of the frontal cortex including premotor areas of the frontal lobe (Maier et al., 2002; Lemon and Griffiths, 2005; Lemon, 2010). The corticomotoneurons in the primary motor cortex (M1) are the major source of descending motor commands for voluntary movement (Lemon, 2010; Quallo et al., 2012; Vigneswaran et al., 2013). These originate, in part, from CST neurons in cortical layer V, whose axons descend to the spinal cord. CST neurons are of two general types. In one type, axons terminate in the intermediate zone of the spinal cord, where they contact spinal interneurons. Some of these interneurons make connections with spinal motoneurons and mediate a component of the descending commands for movement (Maier et al., 2002). The axons of the second type of CST neuron terminate in the ventral horn of the spinal cord, where they make *monosynaptic* connections with spinal motoneurons. These CST neurons are termed cortico-motoneuronal cells, and are thought to have a role in the generation and control of highly skilled movements, including skilled distal movements and the independent use of digits (Muir and Lemon, 1983; Baker et al., 1995). However, spinal motoneurons receive synaptic input from many sources, implying that even direct corticomotoneurons have a variable influence on the muscles they innervate. Single motor tract axons are not a simple “private line” connecting the cells of origin and spinal motoneurons innervating a single muscle, but instead they may exert simultaneous excitatory and inhibitory influences on different groups of spinal interneurons and motoneurons of multiple muscles at widely separated spinal segments (Shinoda et al., 1986, 2006) as part of the requirement for motor control – see **Table 1**.

**Table 1:** Some characteristics of descending motor tracts.

**(1) The lateral descending motor tract group(Cortical spinal and rubrospinal tracts):**
Run mainly in the contralateral lateral funiculus of the spinal cord.
Mainly control distal limb muscles rather than axial and proximal muscles.
Exert stronger excitatory effects on flexor muscles and stronger inhibitory effects on extensor muscles.
Excitatory inputs are mediated monosynaptically and inhibitory inputs disynaptically.
**(2) Medial descending motor tract group (Vestibular spinal tract, tectospinal tract, reticulospinal tract).**
The medial system is phylogenetically and ontogenetically older than the lateral system.
The tracts in the medial system mainly run in the ventral funiculus.
The medial system characteristically steers the body, and integrates limb and body movements as well as developing movement synergisms of individual limbs, involving various parts.
Discrete lesions of the medial system usually produces motor disturbance of the axial and the proximal muscles.

When ALS symptoms begin in the upper limb, typically it is the pincer (precision) grip that is initially weak. The resulting “split-hand syndrome” in which there is thenar-hand weakness and wasting (thenar complex and first dorsal interosseous), with relative sparing of the hypothenar hand, is characteristic of ALS (Eisen and Kuwabara, 2012; Menon et al., 2013). TMS studies of the size of the excitatory postsynaptic potential (EPSP) recorded from the first dorsal interosseous muscle (FDI) during different hand tasks demonstrate the largest EPSP when the FDI is used in a pincer grip (Flament et al., 1993). Whereas in normal subjects the cortical:peripheral ratio (motor evoked potential/compound muscle action potential amplitude) is larger for the thenar compared with the hypothenar complex, in keeping with a stronger corticomotoneuronal input to the thenar hand, this ratio is reversed in ALS, indicating selectively reduced corticomotoneuronal input to the thenar complex (Weber et al., 2000). It is possible that increased functional connectivity, determined by resting state functional MRI, in both the sensorimotor and frontotemporal systems in ALS may reflect a compensatory process in relation to the structural breakdown of the motor and frontotemporal systems (Agosta et al., 2013; Filippi et al., 2013).

During primate evolution a reduction in the number of synapses between the motor cortex and spinal motoneurons innervating the digits has occurred, with an extension of the direct neocortical, corticomotoneuronal projections beyond the cervical segments of the spinal cord (Lemon and Griffiths, 2005; Vigneswaran et al., 2013). Magnetic stimulation studies have shown that in humans there are direct monosynaptic connections from the motor cortex to the motoneuron pools of virtually all muscle groups, except those of the extra-ocular muscles and vesical and anal voluntary sphincter muscles, and the abductor muscles of the larynx, which are uniquely less vulnerable in ALS until late in the course of the disease (Eisen and Weber, 2001). Reaching and grasping, actions that are fundamental to prehension and the manipulation of objects and tools in primates are complex coordinated activities that are particularly sensitive to abnormalities in central motor control (Lemon and Griffiths, 2005). For example, in the macaque, corticomotoneuronal cells can be just as active during tool use as during precision grip (Vigneswaran et al., 2013).

In humans and other primates, phylogenetically older, indirect pathways project from the motor cortex onto motoneurons (Lemon and Griffiths, 2004). These pathways include the propriospinal, reticulospinal, and rubrospinal tracts (see **Table 1**). These phylogenetically, older indirect pathways project to segmental interneurons but their contribution to hand movements remains uncertain (Lawrence and Kuypers, 1965, 1968a,b; Isa et al., 2013).

### CORTICOMOTONEURONAL HYPEREXCITABILITY

Eisen et al. (1992) suggested that corticomotoneuronal hyperexcitability might be a mechanism causing both UMN and LMN degeneration in ALS through glutamate-induced excitotoxicity. Increased plasma glutamate levels were first recognized by Plaitakis and Caroscio (1987). TMS studies have demonstrated that cortical hyperexcitability is an early feature of sporadic and familial ALS, linked to motoneuron degeneration (Vucic and Kiernan, 2013; Vucic et al., 2013). In addition, longitudinal studies in asymptomatic SOD-1 mutation carriers and in the G93A SOD1 mouse model (Browne et al., 2006), revealed that cortical hyperexcitability develops prior to the clinical disease onset (Vucic et al., 2008). Loss of parvalbumin-positive inhibitory interneurons in the motor cortex of ALS patients probably contributes to the development of cortical hyperexcitability (Nihei et al., 1993). In addition, reduced expression of the astrocytic glutamate transporter, excitatory amino acid transporter 2 (EAAT2), has been reported both in the SOD-1 mouse model and in the motor cortex and spinal cord of ALS patients (Philips and Rothstein, 2014).

An increased expression of glutamate receptors permeable to excessive influx of Na^+^ and Ca^2+^ ions has been reported on motoneurons in ALS, thus increasing susceptibility to glutamate toxicity (Williams et al., 1997). There are several molecular features which may render motoneurons vulnerable to glutamate toxicity in ALS. First, motoneurons preferentially express glutamate receptors, such as the AMPA receptors, which are more permeable to influx of Ca^2+^ ions (Kawahara et al., 2004), and motoneurons in ALS patients lack the intracellular expression of Ca^2+^ binding proteins parvalbumin and calbindin D28k, both required to buffer intracellular Ca^2+^ (Alexianu et al., 1994). Aberrant activity of the inositol 1,4,5-triphosphate receptor type 2 receptor has been reported in ALS resulting in higher intracellular concentrations of Ca^2+^ within the motor neurons (Choe and Ehrlich, 2006). Ultimately, an influx of Ca^2+^ ions through the ionotropic glutamate receptors NMDA occurs, resulting in increased intracellular Ca^2+^ concentration and activation of Ca^2+^-dependent enzymatic pathways that mediate neuronal death (Choi, 1987; Meldrum and Garthwaite, 1990). Glutamate excitotoxicity may also result in production of free radicals and thereby cause cell death (Bondy and LeBel, 1993; Bondy and Lee, 1993).

### CORTICOMOTONEURONAL FIRING

Motor maps derived using intracortical microstimulation have suggested that the motor cortex consists of a mosaic of individual columns, each controlling a single muscle (Asanuma, 1975). However, multiple columns of cortical motoneurons, distributed relatively widely in the motor cortex innervate single spinal motoneurons (Rathelot and Strick, 2006); and individual CST neurons target multiple LMNs representing both agonist and antagonist muscles (Cheney et al., 1985). As in sensory maps, motor maps consist of clusters of functionally related neurons, but their topographic arrangement is coarser (Schreiner and Winer, 2007). Movements can most easily be evoked by stimulation of the deep layers of the motor cortex (Young et al., 2011). Recovery after a lesion in the motor cortex reflects re-establishment of patterns of neuronal firing and cortical circuitry. Maps of movement categories, and the corresponding firing patterns of individual neurons in the motor cortex can be related to patterns of movement (Melgari et al., 2008; Zartl et al., 2013), a hypothesis first suggested by Hughlings Jackson, and demonstrated by cortical stimulation in the macaque by Ferrier in the 19th century. Penfield and colleagues in Montreal, later showed this to be true also for the human brain. Individual CST neurons generally influence whether facilitatory or inhibitory LMNs innervating groups of muscles – termed the cell’s muscle field (Aflalo and Graziano, 2006a).

Microstimulation studies established the sufficiency of motor cortical activity in directing movement and suggested that semidiscrete subregions control the movement of distinct body parts (Rasmussen and Penfield, 1947; Donoghue and Wise, 1982; Gioanni and Lamarche, 1985). This topographic organization, however, reveals relatively little about the precise operations performed by motor cortical networks (Aflalo and Graziano, 2006b). Studies using longer-duration stimulation in monkeys and mice raise the possibility that the motor cortex may be more accurately subdivided on the basis of their involvement in different categories of behavior – defensive postures or movements of the hand to the mouth are examples (Harrison and Murphy, 2012; Bonazzi et al., 2013; Griffin et al., 2014). Movement tuning is defined by the firing rate of component neurons. It must ultimately arise from the pattern of input that drives a particular neuron to fire. As in other cortical areas, local inhibitory neurons in motor cortex act in concert with excitatory neurons to tune downstream cells. Electrophysiological recordings have found that fast-spiking interneurons in motor cortex contribute to movement tuning by restricting all but the most excited neurons from firing. Inhibitory neurons increase their firing rates throughout movement preparation and execution, so they are probably involved in shaping movements. Dendritic gating, and amplification, may provide additional modulation (Aflalo and Graziano, 2006a). The firing rate of a single motor cortex neuron is coarsely related to the direction of arm movement, but the activity of a population of neurons can be transformed into a vector that governs the speed and direction of arm movement.

In ALS, positron emission tomography studies indicate greater cerebral blood flow during motor activation in the contralateral primary sensorimotor cortex and adjacent ventral premotor and parietal association cortex, lateral premotor cortex, the supplementary motor area, the anterior cingulate cortex, the paracentral lobule and the superior and inferior and inferior parietal cortex (Kew et al., 1993). The enhanced motor activation with more marked involvement of the premotor cortex was later documented by functional resonance magnetic studies (Konrad et al., 2002). This observation probably results from cortical reorganization in response to Betz cell loss (Kew et al., 1993).

## LOWER MOTOR NEURON AND MOTOR UNIT RECRUITMENT

The LMN consists of the spinal or brainstem motoneurons that receive input from the descending CST system, as well as from other descending and local segmental connections, many of them interneuronal or conveying afferent input, together with their axonal connections to the cluster of muscle fibers innervated by each motoneuron. The number of muscles fibers making up a motor unit varies in different muscles, with the smallest number in muscles such as finger intrinsic muscles that are engaged in delicate coordinated movement. They show homogeneous histochemical patterns in each motor unit related to their contraction and relaxation characteristics, broadly classified as type I, Type IIA or type IIB motor units (Burke et al., 1971).

In each muscle, force may be graded by varying the firing rate of the activated motor units. The relation between force and firing rate follows a sigmoid curve, force gradation takes place within the middle steep portion of this curve (Kernell et al., 1999).

### MOTONEURONS AND THEIR CONNECTIONS

The control of movement can be evaluated at several levels of the motor control hierarchy, but it is ultimately the spinal motoneuron that is the “lens” through which all motor circuitry must focus its activity (Miri et al., 2013). In general, afferent sensory, efferent motor and interneuronal inputs will dictate LMN firing and so influence movement.

### SPINAL MOTONEURON FIRING

The conventional view regarding LMN firing is that during maintained low intensity motor activities, spinal motoneurons fire at a rate that in healthy humans is greater than 5–6 Hz, with inter-spike latencies which are not fixed, but which fluctuate to a limited degree. From extensive work on feline and rat LMNs and modeling studies in humans, it is believed that during motor activity voltage trajectories of mammalian motoneurons, including human LMNs, display predictable changes in firing frequency (Kernell, 1965; Schwindt and Calvin, 1972). Kernell (1965) claimed that LMN firing rates in cat increased linearly with stimulating current until the firing rate reaches approximately 25 Hz (so called “primary range” firing). With stronger stimulation, firing rate increases linearly in many LMNs, but with a greater slope in the relation of stimulating current and firing frequency (so called, “secondary range” firing; Kernell, 1965). In cat LMNs, secondary range firing can reach rates in excess of 125 Hz (Kernell, 1965). A major determinant of the firing rate is the change in membrane voltage following the spike, the after-hyperpolarization potential (AHP). This waveform has both a descending component (“the scoop”), and an ascending, gradually depolarizing component (“the ramp”), when the membrane potential rises to spike threshold. Slow motoneurons exhibit AHP half-decay times longer than 20 ms (Gossen et al., 2003; Button et al., 2006). However, in humans at low-firing rates (“subprimary range”) LMN depolarization is driven by the noisy input (Matthews, 1996; Kudina, 1999; Macdonell et al., 2008). Adaptation and steady-state firing in motoneurons are assumed to be governed by summation of AHP conductance, the amount of adaptation and the shape and slopes of the steady-state frequency-to-current relation can be explained by non-linear summation of successive spikes (Baldissera et al., 1978).

Multiple potassium conductances contribute to repolarization and therefore the AHP of the LMN action potential, some with short duration “fast” components mediated by IA (4-amino-pyridine-sensitive) and IK (tetraethylammonium-sensitive) potassium channels. “Medium”-duration components of the AHP are mediated by Ca^2+^-dependent K^+^ conductances (medium afterhyperpolarization; e.g., apamin-sensitive conductance; Zhang and Krnjevic, 1987; Viana et al., 1993), which are generated by small-conductance calcium-activated potassium channels (Sah and Faber, 2002; Miles et al., 2005; Deardorff et al., 2013). Differences between the AHP in LMNs of different sizes (small and large) and between LMN types (fast, F also called type II; and slow, S, also called type I; there are also fatigue-resistant type IIa and fast-fatigable type IIb MNs) are partially responsible for the different firing characteristics of these LMNs. The medium-duration AHP is mediated by the apamin-sensitive, SK channel subunits SK2 and SK3, where SK2 is present in all rat alpha-MNs and SK3 is expressed only in a population of S-type rat MNs with longer duration, larger amplitude AHPs (Deardorff et al., 2013). SK2 and SK3 channels are present in feline LMNs in which the peak of the AHP amplitudes appears to be related to the proportion of SK3 and SK2 channels (Deardorff et al., 2013). These SK2 and SK3 channels are prominently associated with cholinergic C-type synapses, a potential site of modulation of AHP duration and hence, LMN firing rate (Deardorff et al., 2013). Computer simulations of the voltage trajectories of LMNs have been used to derive similar biophysical properties of cat and human LMNs (Jones and Bawa, 1997). Sites of modulation of LMN firing rate include inputs from CST inputs, muscarinic input from interneurons, monoaminergic projections and others especially afferent projections.

Although there is a limited understanding of the relation between LMN firing and the biochemical properties of LMNs recent studies have begun to identify biochemical profiles of different LMN populations. For instance, the notch protein signaling pathway appears to be relevant for LMN specification. In vertebrates and invertebrates neuronal fate is influenced by this pathway which simplistically includes a receptor termed “notch” and a ligand, often called “delta,” where both delta and notch can be transmembrane proteins which influence cell–cell interactions. The notch ligand, delta-like homolog 1 (Dlk1) is expressed more highly by large LMNs, compared to small LMNs and co-relates with the fast firing IIb LMN phenotype both by LMN size and firing frequency (Muller et al., 2014). Although Dlk1 may “promote a fast LMN biophysical signature,” the variability in expression of Dlk1 is also consistent with previous views that LMN properties may be distributed continuously within the LMN population (Heckman and Enoka, 2012), perhaps related to the variability in expression of specific biochemical “markers” such as Dlk1. Dlk1 could modify LMN properties by changing the profiles of genes expressed in the Dlk1+ expressing MN population. One of the genes modified by Dlk1 expression is a subunit of the delayed rectifier K+ conductance in LMNs called Kcng4 which may be responsible for some but not all of the changes in firing frequency seen in the type IIb LMNs (Muller et al., 2014).

The properties of LMNs profoundly influence their susceptibility to development of ALS. It has been known from studies in mice over-expressing mutant human SOD1 that fast fatiguing (type IIb) LMNs are much more susceptible to LMN axon loss from muscle targets than type I LMNs (Frey et al., 2000; Pun et al., 2006; Hegedus et al., 2008; Saxena et al., 2013). In longitudinal studies of isometric twitch forces and immunocytochemical assessment of muscle innervation in mouse it has been determined that there is impaired sprouting ability of the larger, fast fatiguing IIb LMNs and that axonal die-back of LMNs precedes a significant decline in LMN number (Kennel et al., 1996; Fischer et al., 2004).

A consequence of the stereotyped voltage trajectories of LMNs is the relatively regular firing pattern of normal LMNs and the belief that some specific impairment of LMN firing might characterize ALS. Previous studies have reported irregular firing in single motor unit potentials (MUPs) in ALS patients (Piotrkiewicz and Hausmanowa-Petrusewicz, 2013), when other simultaneously recorded MUPs from the same muscle fired regularly. These observations were interpreted as suggesting that disease involvement occurred disproportionately in some LMNs, compared to others. The irregularities of firing are attributed variably to ectopic discharges, two or more sequential firings of a MUP (double discharges or “doublets”), or the blocking of neurotransmission in small axonal branches (Piotrkiewicz and Hausmanowa-Petrusewicz, 2013). The loss of LMNs typically results in an increase in the MUP firing rate with an “incomplete” recruitment pattern during routine EMG recordings. However, there is also often a reduction in the firing rate of MUPs with sustained contractions which has often been labeled as “fatigue” and is poorly understood.

The characterization of changes to LMN excitability in ALS has proved challenging. One approach to defining altered LMN excitability is to employ a technique called “threshold tracking” (Burke et al., 2001), which examines nerve excitability by applying subthreshold current pulses and monitoring voltage responses to track changes in the threshold by which modulation of the underlying ionic conductances can be inferred. This method is described elsewhere and has led to claims that ALS patients have altered Na^+^ and K^+^ conductances depending on the stage of disease (Kanai et al., 2006). Early in ALS, patients may have increased persistent Na+ conductances which could lead to the appearance of fasciculations. Later in the disease, K+ conductances decline (Kanai et al., 2006). However, electrophysiological changes found in ALS patients are not replicated with similar protocols applied to mice over-expressing human mutant SOD indicating differences between ALS and the mouse model (Boerio et al., 2010).

Clinically or electrophysiologically detectable fasciculations are almost universal in ALS, but are not requisite for diagnosis. Fasciculation refers to the spontaneous, intermittent activation of some or all of the muscle fibers of one or more motor unit. Isolated fasciculation potentials, without other neurogenic changes are observed in early affected muscles of ALS patients, before increased “jitter” and denervation signs are observed (de Carvalho and Swash, 2013a). The relatively early onset of fasciculation in ALS has led to the view that it results from MN excitability and is a marker or consequence of the MN dysfunction (Denny-Brown and Pennybacker, 1938; de Carvalho and Swash, 2013b). However, fasciculations are not specific in that they can be recorded in many diseases that affect MNs and are often observed in healthy individuals. There is also evidence that some fasciculations are generated at supraspinal sites (de Carvalho et al., 2000). Specific ion channel conductances that might be impaired in ALS include those related to mAHP duration and have long been believed to be a potential factor modulating LMN firing rate. For instance, in experiments using axotomized MNs, Kuno et al. (1974), found changes in mAHP durations which they postulated might be related to a “trophic” signal associated with the activity of the innervated muscle retrogradely transported to the LMN soma (Czeh et al., 1978) or to increased activation of “C”-type synapses on LMN somata as a compensatory response to LMN de-afferentation (Pullen and Sears, 1983). How LMNs function as part of neuron circuits or as members of a more widely distributed “connectome” is a key issue for future research.

### CORTICOSPINAL INPUT TO MNs

The differing patterns of corticospinal tract projections in different species may correlate with the susceptibility to LMN dysfunction in some species and not others. The relation between CST projections and LMN dysfunction is discussed above – see **Tables 1 and 2**.

**Table 2:** Some descending and segmental inputs on motor neurons.

Tract or Input	Origin	Target	Significance	Reference
Corticospinal tract	Widespread cortical regions – especially frontal cortex	Direct contact with dendrites of motor neurons	Single CST axons project to different motor neuron pools	Maier et al. (2002),Lemon and Griffiths (2005),Lemon (2010)
Rubrospinal tract	Magnocellular red nucleus	Motor neurons of forelimbs	Significance unknown, possible role in early motor development	Humphrey et al. (1984),Cheney et al. (1985)
Reticulospinal tract	Arises from pontine and medullary reticular formation	Direct and indirect inputs to motor neurons	May influence motor behavior especially after damage to CST.Regulate tonic motor neurons	Wilson and Yoshida (1969), Riddle et al. (2009),Baker (2011)
Propriospinal tract	Cervical spinal cord	May be indirect	Reaching and grasp behavior,may mediate some CST activation	Kinoshita et al. (2012)
Locus coeruleus	Fibers arise from locus coeruleus, but also adjacent brainstem sites	Widespread projections in spinal cord including motor neurons	Important source for adrenergic inputs to motor neurons, may regulate persistent inward currents (PIC)	Kuypers (1981),Lee and Heckman (1998), Heckman and Enoka (2012)
Raphe nucleus	Raphe nucleus	Widespread projections	Likely provide serotonergic input to motor neurons	Kuypers (1981)
Voc neurons	Small group of spinal interneurons (transcription factor Pitx2)	Direct input to motor neuron soma and proximal dendrites (∼5%)	Responsible for cholinergic input to motoneurons and “C-type” synapses	Zagoraiou et al. (2009)
Other interneuron populations	Other small interneuron populations (V1–V3)	Segmental inputs	Modulation of motor neuron firing	Zagoraiou et al. (2009)

### CHOLINERGIC INPUTS: THE C-TYPE SYNAPSE

The distinctive C-type synapses on LMNs exhibit presynaptic, pleomorphic vesicles, and a post-synaptic submembranous cisternal structure (Pullen and Sears, 1983; Pullen and Athanasiou, 2009). These synapses comprise 5–8.5% of the total synaptic content on LMNs and are particularly abundant on soma and proximal dendrities. C-type synapses are associated with muscarinic Ach receptors (mAchR; m2), as well as with K channels (Kv2.1) and SK channels. They are resistant to de-afferentation and spinal hemisection, suggesting that they represent a specific population of spinal cholinergic interneurons. Zagoraiou et al. (2009) have shown that the sole source of C-type synapses is a small group of spinal interneurons (VOc neurons) that express the transcription factor Pitx2. The somata of these cholinergic neurons are located around the central canal and are particularly associated with innervation of LMNs supplying proximal than distal muscles. These interneurons are associated with locomotion (Zagoraiou et al., 2009). They modulate LMN excitability by regulating the mAHP, presumably through their association with SK channels.

These C-type inputs may be particularly important for our understanding of ALS. Examination of LMNs in a transgenic mouse model of ALS and in patients dying with ALS, have shown an increase in C-terminal coverage of spinal LMN with disease progression (Pullen and Athanasiou, 2009). It has been suggested that this is a compensatory response to loss of descending inputs in ALS. Moreover, it is likely that increased C-terminal input will modify LMN firing properties. The relevance of this adaptive response may explain, in part, the clinical observation as to why there are not more prominent UMN signs in ALS patients, even in the presence of substantial UMN loss (Swash, 2012).

### MONOAMINERGIC DESCENDING PATHWAYS

Reticulospinal projections probably contribute to LMN excitability through monoaminergic pathways, including serotonergic and nor-adrenergic fibers (Heckman and Enoka, 2012). These monoaminergic projections largely synapse with LMN dendrites and their activation results in large, persistent inward currents.

### THE DOUBLE DISCHARGE (DOUBLET)

During the evaluation of patients by concentric needle EMG, MUPs are sometimes seen to fire in groups of two or three. Double discharges are rarely found in normal individuals, although they can observed in some muscles during slow voluntary contractions in trained subjects (Bawa and Calancie, 1983; Kudina and Andreeva, 2013). In healthy subjects, the motoneurons with early excitability recovery are capable of firing double discharges with a 5–15 ms interspike interval (Kudina and Alexeeva, 1992). The incidence of double discharges is significantly higher in ALS patients, but doublet interval durations and firing patterns of doubling motor units did not differ between ALS and healthy subjects (Piotrkiewicz et al., 2008). It might represent dysfunction of the LMN pool in ALS (Zalewska et al., 1998). The underlying mechanism should involve delayed depolarization (Weber et al., 2009; Kudina and Andreeva, 2010).

### THE PRESYNAPTIC TERMINAL OF THE MOTONEURON

Most neurodegenerative diseases are characterized by extensive neuronal dysfunction and death within the central nervous system. The functional deficit in ALS has therefore been assumed to follow neuronal death. However, there is a recent literature claiming that neuron pathology in ALS is due to a degenerative process that begins in the presynaptic terminal, the neuromuscular junction, or the distal axon (Fischer et al., 2004), which is described in normal aging (Valdez et al., 2012). This degenerative process may initially lead to dying back of the distal axon (“distal axonopathy”), with relative histological preservation of the neuronal cell body and proximal axon. This “dying back” process causes dysfunction in the longest and largest motor axons first. Subsequently, more proximal portions of the axon become affected. This view is based on neuropathological evaluation of some types of toxin-induced peripheral nerve damage (peripheral neuropathy), in which the small intramuscular nerve branches may be affected initially but more proximal portions of axons are spared. This phenomenon probably results from metabolic changes in distal parts of axons associated with reduced axonal transport.

In ALS patients, and in a rodent model of ALS based on over-expression of mutant human superoxide dismutase (mSOD), derived from familial human ALS, pathological changes consistent with distal axonopathy have been reported. Similar changes have been recognized in human familial mutant SOD1 ALS (Fischer et al., 2004). In addition, changes in neuromuscular transmission, presumptively related to changes in pre-synaptic Ca^2+^ concentration, or altered calcium channel function have been observed (Appel et al., 2001). In the transgenic mouse model a large proportion of neuromuscular junctions show denervation change at a stage when there are only slight reductions in in the numbers of spinal LMNs or ventral root fibers, as determined by electrophysiological (Kennel et al., 1996), or morphological assessment (Fischer et al., 2004; Parkhouse et al., 2008). Furthermore, there are differences in the susceptibility to degeneration of various synaptic subtypes. Synapses of fast fatigable LMNs are vulnerable to synaptic loss, whereas the synapses of slow LMNs are relatively resistant (Frey et al., 2000; Pun et al., 2006; Hegedus et al., 2008). These progressive changes in specific synapses with progressive disease suggest that synapse-specific mechanisms are important. This view is supported to some degree by the observation that the WldS gene, which protects against axonal injury, modestly prolongs survival in the mSOD mouse (Fischer et al., 2005). Synaptic terminals that are less susceptible to denervation also demonstrate greater ability to generate stimulus-induced synaptic sprouting. This might arise from differences in the regulation of the actin cytoskeleton of the synapse. It is also possible that modulation of presynaptic autoreceptors on LMNs might influence its function. For instance, protein kinase alterations influence LMN neurotransmitter release (Santafe et al., 2006). Collectively, these observations suggest that the physiological properties of motor neurons influence firing and that these firing properties can, in turn, influence motor neuron survival, likely by modulating cell excitability (Saxena et al., 2013).

### FUNDAMENTAL CURRENT ISSUES

There has been long standing interest in the possibility that the ionic, or ligand-gated channels expressed by motor neurons might be associated with vulnerability to dysfunction or death in ALS. Considerable attention has been paid to the role of ligand-gated excitatory amino acid receptors, with regard to excitotoxicity, and this topic has been reviewed extensively. Although the mechanisms that lead to aberrant firing of motor neurons, such as the generation of fasciculations in ALS, are unclear, it is clear that motor neuron action potential generation remains critical even as the disorder progresses. As described above, the AHP and conductances underlying this phase of the action potential are modulated by cholinergic inputs (Heckman and Enoka, 2012), and these synaptic inputs are abnormal in a murine model of ALS (Fischer et al., 2004). Nonetheless, involvement of descending inputs from cortex, or from subcortical structures such as monoaminergic axons from locus coeruleus (noradrenergic), or the median raphe (serotonergic), which facilitate persistent inward currents could underlie hyper-excitability and cell dysfunction, and cell survival. Axonal and presynaptic dysfunction is also relevant to the development of ALS. Furthermore, although it is beyond the scope of this review, it is important to recognize that cell dysfunction is not restricted to motor neurons, since other cells in the vicinity of UMNs or LMNs, such as astrocytes, microglia, or glia in the axon periphery may contribute to the development of ALS.

## MOTOR UNIT FIRING IN NEUROLOGICAL DISEASE

Motor unit recruitment and firing patterns determine the characteristics of individualized muscle and limb functions; this requires organization at cortical, basal ganglia, cerebellar, and spinal level in a complex and interwoven output, based on hardware (connections and pathways) and software (learned patterns and combinations of activities). Much remains to be learned about the mechanisms that determine functional capacity.

The main EMG feature in ALS is the abnormal MUP recruitment derived from LMN loss, in particular we observe a reduced number of recruited motor units and an increased firing rate of the recruited motor units. The recruitment order is probably not much altered in ALS (Milner-Brown et al., 1973), but it has been suggested that the recruitment order can be abnormal in neurogenic atrophy, with motor units generating high-twitch tensions sometimes recruited first (Herdmann et al., 1988). Physiologically, the firing rate of new recruited motor units tend to stabilize when other units are added to the recruitment pattern. Indeed, a slight increase in the firing rate is observed in the initially recruited units, but the primary mechanism for increasing force output is the spatial recruitment of more motor units (Petajan, 1991). When no more motor units can be recruited firing rate is very increased, up to 50 Hz. ALS combines both lower and upper motoneuron involvement in the same segment. The impact of UMN lesion on the normal fluctuation of LMN discharge rate (Rosenfalck and Andreassen, 1980) is not well known in ALS, but can disturb dexterity and maximal strength.

### MOTOR UNIT ACTIVATION AND ITS MODULATION

The force generated by voluntary striated muscle contraction is regulated by two main mechanisms: recruitment of motor units and modulation of their firing rate. Both recruitment and firing rate modulation arise in response to a common excitatory drive (De Luca et al., 1982). The upper limit of motor unit recruitment differs between muscles, as well as during increasing force. In some hand muscles most motor units are recruited with muscle force less than 40–80% maximum, but in biceps brachii and tibialis anterior are recruited up to approximately 90% maximal voluntary contraction force (Heckman and Enoka, 2012). There are a number of sensory feedback loops that modulate LMN firing rates. The regular discharge of motor units at low rates of firing is modulated by muscle spindle activity, as shown in cat experiments by Burke (1968), and, in human recordings by Hagbarth and Vallbo (1969) and Vallbo (1974). These investigators considered that the spindle discharge reinforced alpha motor neurons activity and was important in sensitizing the motor system to sensory input and therefore in modulating motor activity. Cutaneous afferent input feedback also influences motor unit recruitment (Garnett and Stephens, 1981; Kanda and Desmedt, 1983). After cutaneous stimulation higher threshold units show lower recruitment thresholds, thus altering recruitment order (Kanda and Desmedt, 1983). Low threshold motor unit activity is never entirely regular (Rosenfalck and Andreassen, 1980), especially when a unit is firing very slowly, a feature attributed by Stålberg et al. (1973) to spontaneous fluctuations in the trigger level of the motoneuron membrane, variable presynaptic inflow, and short term irregularity in the neuronal depolarization curve, due to “synaptic noise.” In general, motor units in muscles innervated by lumbar roots show a slower and more regular firing pattern than motor units in upper limb muscles (Stålberg et al., 1973). The variability of low threshold motor unit discharge is higher in old than in young adults, a feature consistent with the greater force fluctuation observed at low muscle forces in older people (Tracy et al., 2005), this probably derives from longer AHP associated with aging (Piotrkiewicz et al., 2007). In addition, discharge rate variability decreases with an increase in muscle force. Adding this change in discharge rate variability to a motor unit model dramatically improved the ability of the model to produce simulated force fluctuations that mirrored those observed experimentally (Moritz et al., 2005). Motor units with a low threshold for maintained voluntary activity show longer contraction times, lower twitch tension, greater fatigue resistance, smaller MUP amplitudes and lower axonal conduction velocity than motor units of higher threshold (Grimby and Hannerz, 1968).

In ALS, the reduced number of motor units is the major determinant of fatigue, but several mechanisms are involved (Thomas and Zijdewind, 2006). The impact of fatigue on the recruitment properties of motor units in UMN lesions, LMN lesions, and mixed disorders is incompletely understood (Bigland-Ritchie et al., 1986). In general, motor unit discharge rate is reduced and is more regular during fatigue (Gandevia, 1998). Several authors have reported silencing of motor units during prolonged contractions (Kato et al., 1981; Fallentin et al., 1993). Several explanations have been proposed: motoneuron adaptation to a constant excitatory input (Kernell and Monster, 1982); reflex dis-facilitation by a decline in the group Ia excitatory input from muscle spindle afferents (Macefield et al., 1991); reflex inhibition from group III and IV muscle afferents (Duchateau and Hainaut, 1993) and modulation of AHP (Person and Kudina, 1972). It has been suggested that a reduction in motor unit discharge rate matches the slowing of motor unit contractile speed during fatigue, optimizes force, and prevents energy loss (Marsden et al., 1983). However, such adaptation might not occur in high-threshold motor units (Carpentier et al., 2001), representing intrinsic differences in motoneuron adaptation to peripheral feedback. Diverse properties of proximal and distal muscles and relative differences in type I and type II muscle fiber proportions in a muscle might explain some physiological differences. In proximal muscles, monotonic decline in the recruitment threshold of the motor units and the progressive recruitment of new motor units without change in the recruitment order was observed during submaximal fatiguing contractions (Adam and De Luca, 2003), but in distal muscles low-threshold motor units show either no change or an increase in recruitment threshold, as opposed to high-threshold ones that tended to show a progressively reduced threshold (Carpentier et al., 2001). However, these dissimilarities could result, in part, from different experimental protocols.

Central drive is intensified during a fatigue task, as shown by the observation of additional higher threshold motor unit recruitment during fatigue tests (Bigland-Ritchie et al., 1986; Garland et al., 1994). However, there is a complex mechanism of motoneuron adaptation and feedback influences (Peters and Fuglevand, 1999).

### IMPACT OF UPPER MOTOR NEURON AND LOWER MOTOR NEURON LESION ON MU RECRUITMENT

In patients with UMN lesions, without LMN involvement, motor units fire more slowly than normal, probably dependent on longer AHPs in LMNs in affected segments. This has been described in stroke (Liang et al., 2010; Ivanova et al., 2014). In ALS, the impact of UMN lesion on AHP of LMN is certainly dependent on LMN dysfunction, it has been described a AHP shortening in early stages of muscle involvement, increasing with higher relative force deficit (Piotrkiewicz and Hausmanowa-Petrusewicz, 2011). This could be explained by the increased susceptibility of the fast motor units to degeneration in ALS (Piotrkiewicz and Hausmanowa-Petrusewicz, 2011; Piotrkiewicz et al., 2012). This explains that in ALS firing rates have been reported as higher than normal in strong muscles but slower in weak muscles (Piotrkiewicz and Hausmanowa-Petrusewicz, 2011; Piotrkiewicz et al., 2012). Moreover, in ALS patients is observed a higher variability of the interspike-interval of the firing motor units, this finding is consistent with increased excitability of the LMN in this disease (Piotrkiewicz et al., 2012).

**FIGURE 1:**
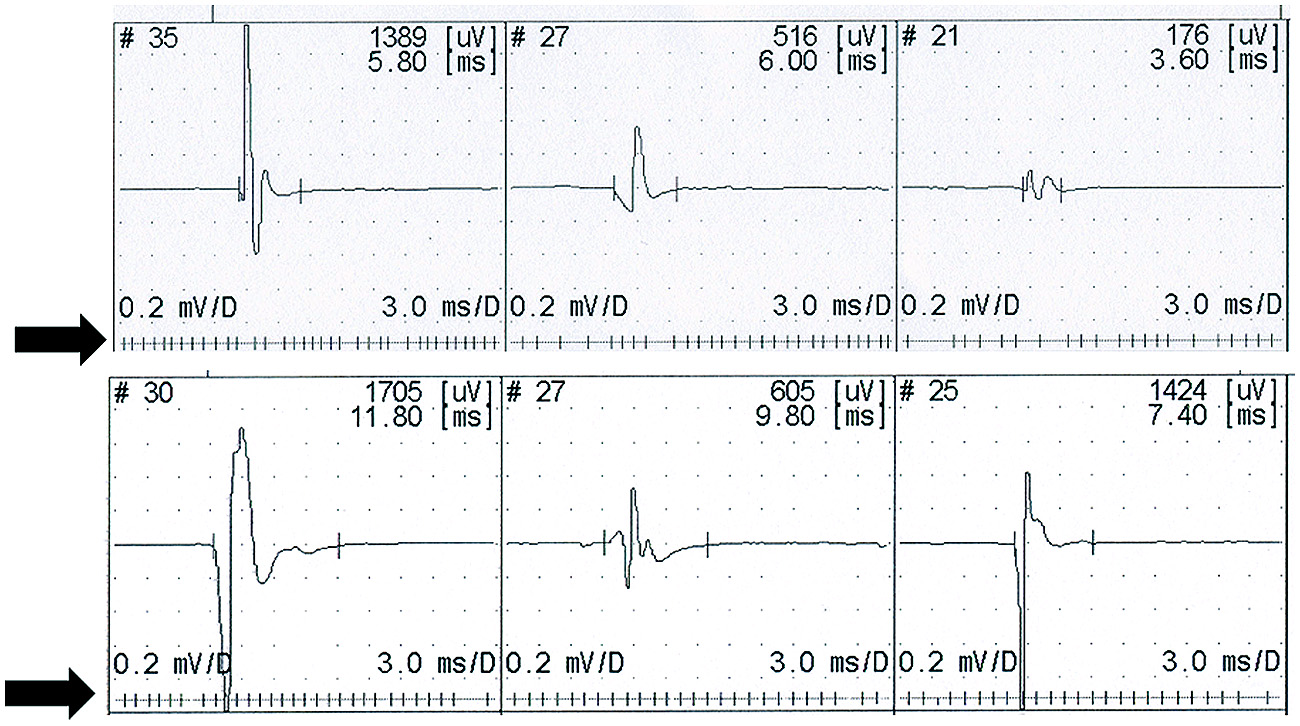
**Recruitment of three motor units on slight contraction.** The upper recording from a control subject shows physiological slight variability in the firing rate. Lower trace shows a stable firing rate recorded in a patient with upper motor neuron lesion (primary lateral sclerosis). Motor unit potentials are displayed above a raster showing the firing rate.

UMN lesion causes a consequent reduction in force (Rosenfalck and Andreassen, 1980), and the normal physiological variability of the motor unit firing rate is reduced (Rosenfalck and Andreassen, 1980; de Carvalho et al., 2012) – **Figure 1**. In patients with ALS there is a complex interplay of different physiological abnormalities within the spinal motor neurons, presumably reflecting the combination of UMN and LMN features found in this disease (Lawyer and Netsky, 1953; Brownell et al., 1970; Chou, 1995), and possibly the involvement of other systems, such as spinocerebellar afferent pathways (Brownell et al., 1970; Swash et al., 1988). Disruption of interneuronal function in the dorso-medial segment of the ventral horn in the spinal cord (Stephens et al., 2006), dysfunction of Renshaw cells (Raynor and Shefner, 1994; Stephens et al., 2006), and loss of gamma motor neurons (Swash and Fox, 1974; Swash et al., 1986), results in loss of local control mechanisms governing motor unit firing patterns at spinal segmental level (Swash, 2012). In ALS patients with marked clinical signs of UMN lesion, the firing rate behavior approaches that found in clinical conditions only affecting UMNs, in muscle with preserved strength (Rosenfalck and Andreassen, 1980). Reduced modulation of LMN firing by peripheral afferents has been reported in PLS (Floeter et al., 2005).

In UMN lesion with spasticity the α-motoneurone is hyperexcitable, so that the membrane potential is closer to threshold than normal, causing facilitation of the effect of voluntary activation on motoneurons, and reduced variability in firing rate. This decreased variability in motor unit firing in UMN syndromes has been explained by activation of persistent inward currents producing stable plateau, which resist changes in response to small inputs (Lee and Heckman, 1998).

In conditions with LMN dysfunction there is a relative failure of the cell’s ability to maintain the membrane potential as close to threshold as in pure UMN disorders. Indeed, in patients with LMN dysfunction, as in polyneuropathy and PMA, there is a trend to increased variability in the mean motor unit firing rate as compared with control subjects (de Carvalho et al., 2012).

## CONCLUDING REMARKS

The complex synaptic relationships between the motor cortex, the descending pyramidal and extrapyramidal motor pathways, including the propriospinal system, and the organization of the spinal segmental motor system itself, are progressively destroyed during the course of ALS (Swash, 2012). This process also involves afferent connections at segmental level. This cascade of degeneration determines the clinical phenotype, but the factors leading to the relative severity of involvement, for example, of cortical neurons, UMN pathways, and LMN systems remain unknown. ALS often begins relatively focally, in that weakness may present in localized fashion in one limb before it spreads, in an orderly mode, to become more diffuse (Ravits and La Spada, 2009). However, there is increasingly convincing evidence that ALS has a long pre-clinical phase, before the disease becomes clinically manifest (Eisen et al., 2014). This phase, however, is currently undetectable except insofar as cortical motor physiological studies, using threshold tracking, have revealed early reduction in cortical inhibition, leading to the concept that motor cortex hyperexcitability may be the earliest detectable effect of the disease, perhaps even preceding LMN dysfunction. The critical abnormality in synaptic input to cortical motor system neurons leading to this effect is uncertain.

A major feature of ALS is the inexorable clinical course once the disease has commenced. There are few, if any, incontrovertible reports of survival, or arrest of disease progression. In this respect, ALS seems particularly malignant in its course. Although ALS has been traditionally regarded as a motor degeneration, there are more widespread changes in the brain; for example, there is early degeneration of the spinocerebellar pathways, a spinal afferent system that synapses in Clarke’s nucleus in the upper cervical region (Swash et al., 1988). Furthermore, involvement of frontal and prefrontal cortex and its connections is a major feature manifesting as fronto-temporal dementia. This is an evident clinical feature affecting more than half of people with ALS. This clinical manifestation can be regarded as functionally part of the effector system of the brain. Posterior brain systems, related to visual, somatic, and auditory sensory functions, and olfaction, and their integrating systems in parietal lobes and spinal cord, are spared. The underlying factors conferring resistance of these pathways to the disease, as is the case also for ocular muscles and the ano-vesical sphincter system, are unknown. With regard to the kinetics of the degenerative process in the motor system, therefore, there is much to be learned.

## Conflict of Interest Statement

The authors declare that the research was conducted in the absence of any commercial or financial relationships that could be construed as a potential conflict of interest.
